# Supplementary material for: The repertoire of resistance mutations selected by a Pseudomonas aeruginosa type IV pilus-targeting lytic bacteriophage
Source: mBio. 2026 Mar 30;17(5):e00243-26. doi: 10.1128/mbio.00243-26 (PMC13170266; doi:10.1128/mbio.00243-26)
Supplement: Supplemental material — Fig. S1 to S6; Tables S1 and S2. [file mbio.00243-26-s0001.docx]

**Supplementary Figures and Tables for:**

**The repertoire of resistance mutations selected by a *Pseudomonas aeruginosa* type IV pilus-targeting lytic bacteriophage**

Veronica N. Tran, Hanjeong Harvey, Tanisha S. Lahane, and Lori L. Burrows^*^

Department of Biochemistry and Biomedical Sciences and the Michael G. DeGroote Institute for Infection Disease Research, McMaster University

*****For correspondence: [lori.burrows@mcmaster.ca](mailto:lori.burrows@mcmaster.ca)

**
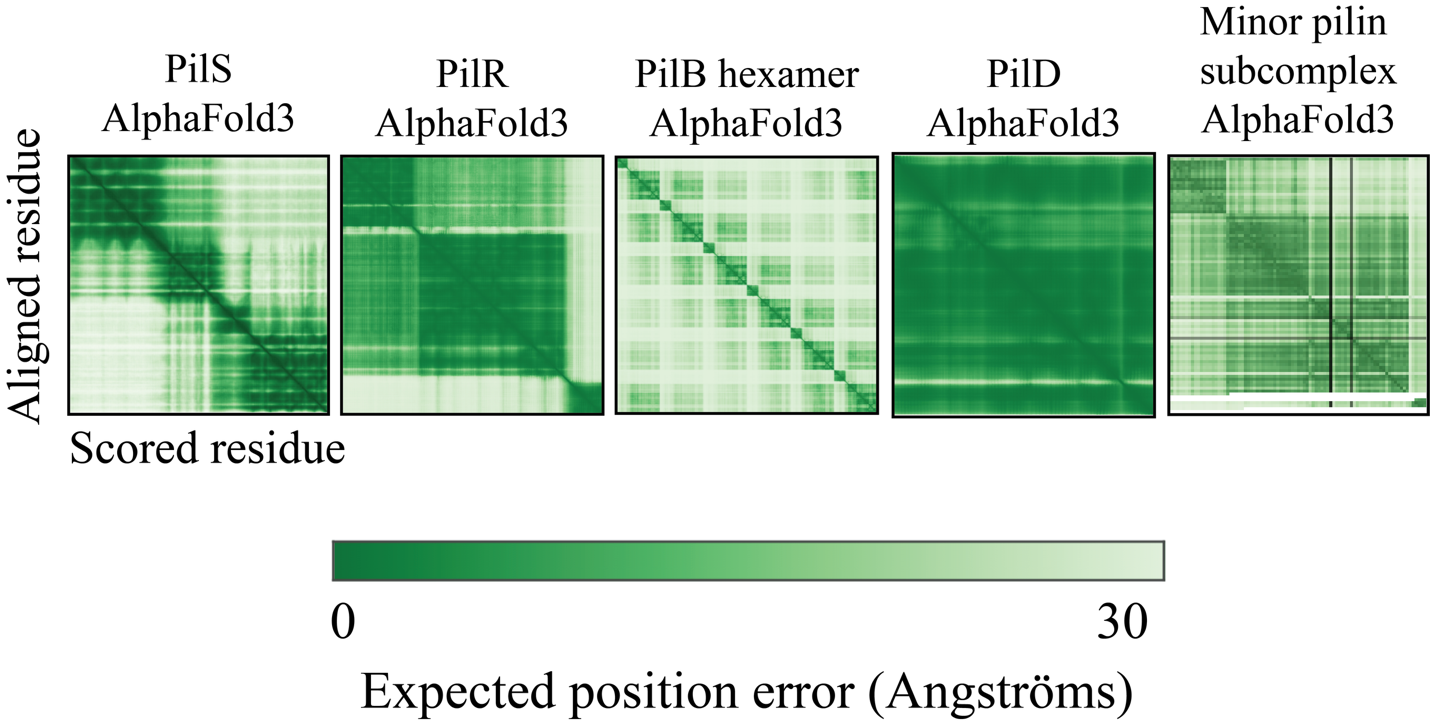
**

**Figure S1. Predicted aligned error plots for AlphaFold predicted structures.** Error plots for predicted structures in **Figure 1** were generated using AlphaFold3 and visualized using UCSF ChimeraX.


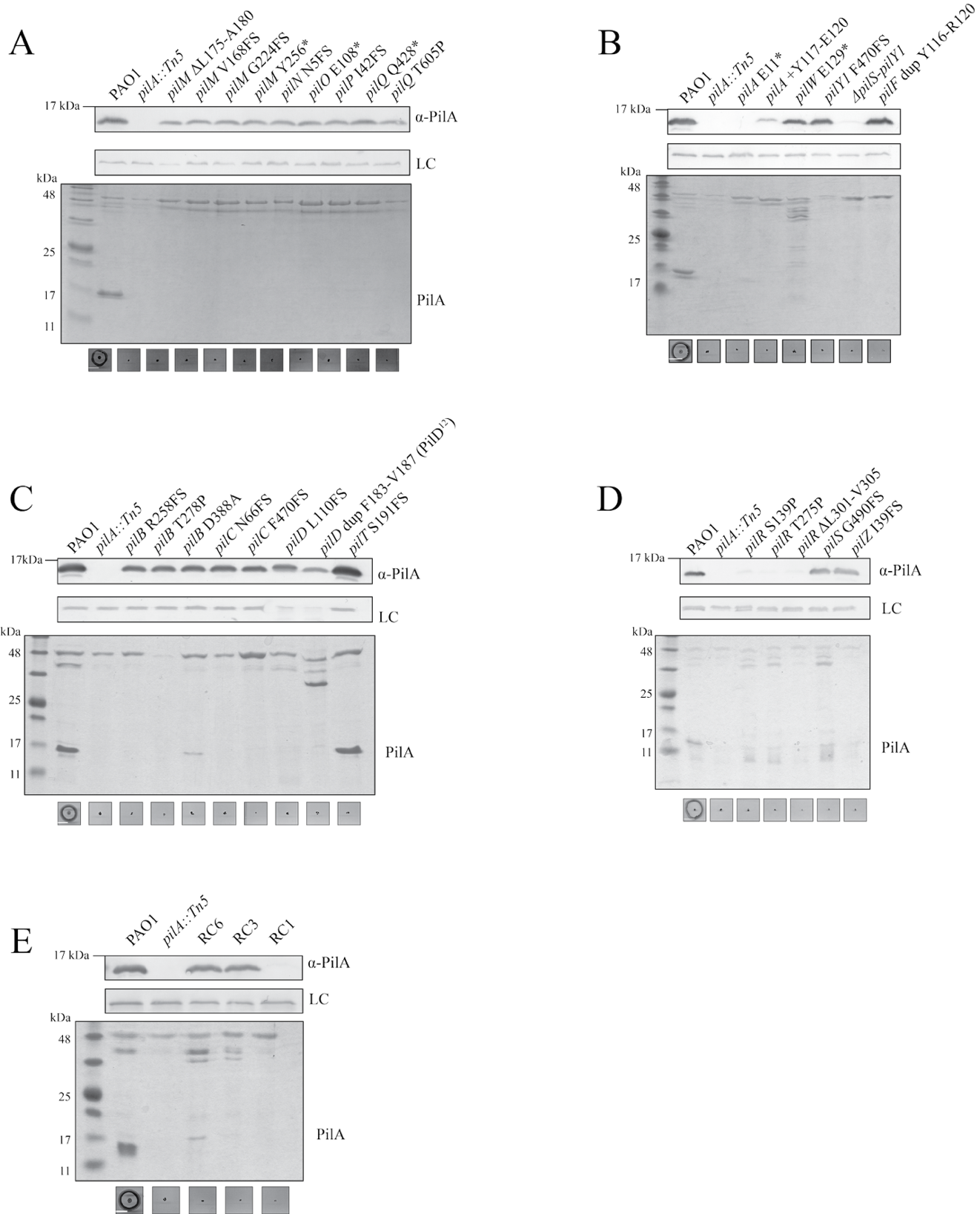


**Figure S2. PRMs do not twitch but have varied pilin production and recoverable surface pilins.** From top to bottom of each panel, Western immunoblots of cell samples representing intracellular pilin production using α-PilA antisera. Sheared surface protein preparations of respective PRMs, analyzed using a Coomasie-stained SDS-PAGE gel. Twitching analysis of each PRM, plates were incubated overnight at 37ºC. PRMs are organized based on mutations in genes of the **A)** alignment complex, **B** major and minor pilins and *pilF*, **C)** inner membrane motor complex, **D)** PilS-R TCS and PilZ, and **E)** representative RANTS. Samples are representative of three independent experiments. Scale bar represents 1cm.


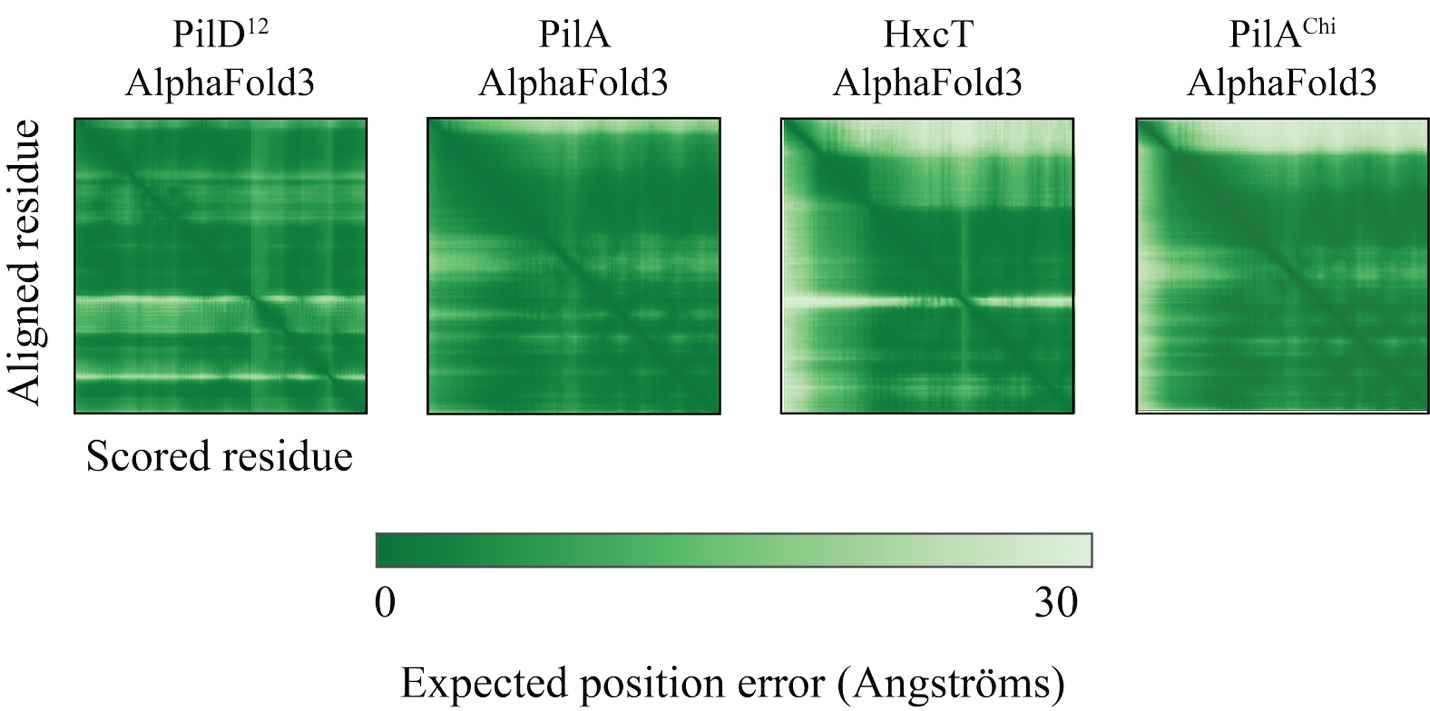


**Figure S3. Predicted aligned error plots for AlphaFold predicted structures.** Error plots for predicted structures in **Figure 3** were generated using AlphaFold3 and visualized using UCSF ChimeraX.


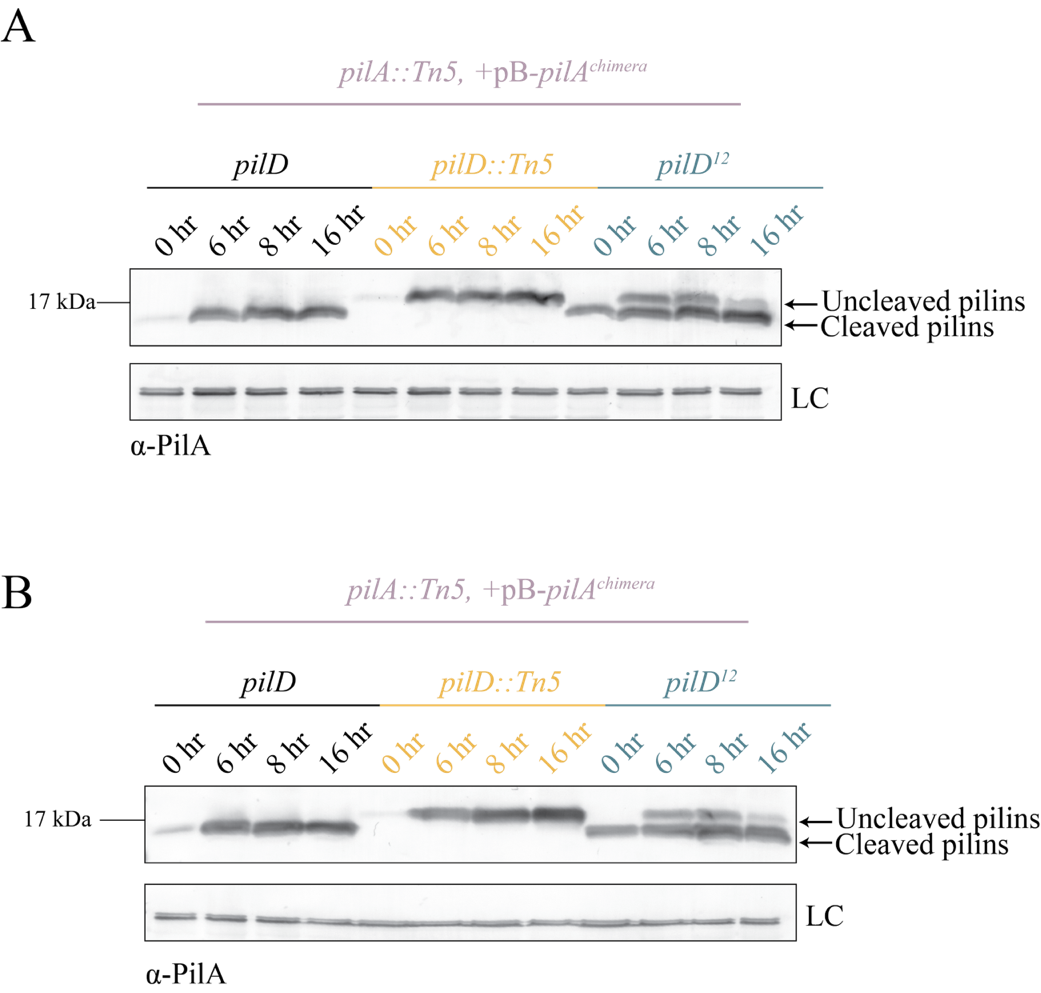


**Figure S4**. **Representative replicates of Figure 3C.** PilD^12^ has more recoverable pilins than WT and NP at initial sample.


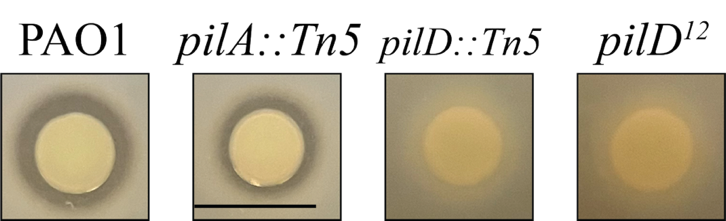


**Figure S5. PilD^12^ protease secretion is impaired.** PilD^12^ spotted on a skim-milk agar plate does not have a visible clearance zone, suggesting lack of T2SS-dependent secreted proteases. Scale bar represents 1cm. Samples are representative of three independent experiments.


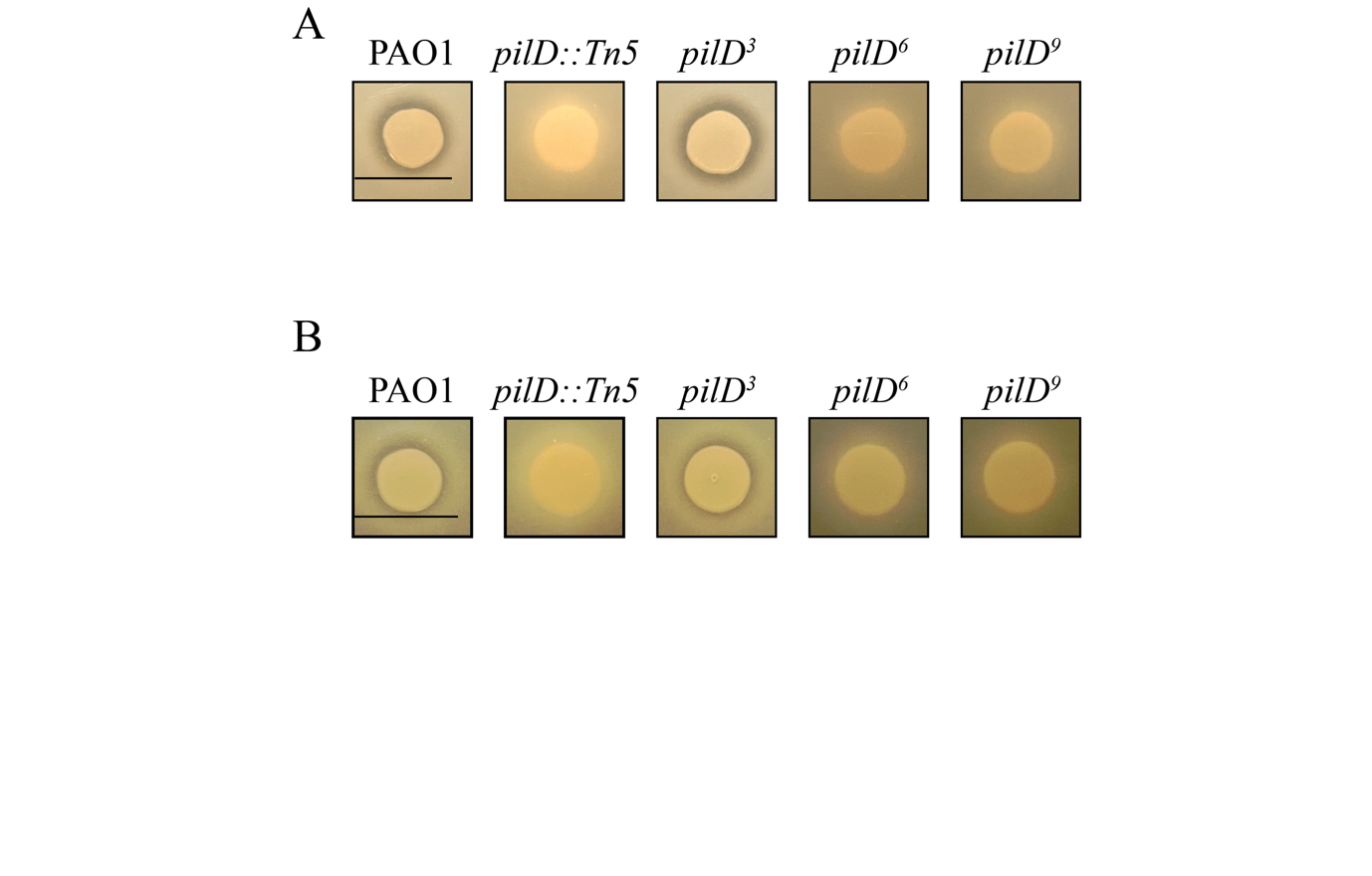


**Figure S6**. **Representative replicates of Figure 6E.** *pilD^3^* spotted on a skim-milk agar plate had a visible zone of clearance, indicating protease secretion.

**Supplementary Table S1. Twitching zone area (cm^2^) if Figure 6B**

| **Strain** | **WT** | ***pilA::Tn5*** | ***pilD::Tn5*** | **PilD^3^** | **PilD^6^** | **PilD^9^** | **PilD^12^** |
| --- | --- | --- | --- | --- | --- | --- | --- |
|  |  |  |  |  |  |  |  |
| R 1.1 | 2.55 | 0.02 | 0.07 | 2.20 | 0.01 | 0.03 | 0.01 |
| R 1_2 | 1.24 | 0.03 | 0.02 | 1.33 | 0.01 | 0.02 | 0.04 |
| Rep 1 avg | 1.89 | 0.02 | 0.04 | 1.77 | 0.01 | 0.02 | 0.02 |
|  |  |  |  |  |  |  |  |
| 2.1 | 2.28 | 0.03 | 0.01 | 1.40 | 0.02 | 0.03 | 0.02 |
| 2.2 | 1.21 | 0.02 | 0.02 | 1.35 | 0.02 | 0.03 | 0.02 |
| 2.3 | 1.67 | 0.02 | 0.01 | 1.39 | 0.01 | 0.02 | 0.05 |
| Rep 2 avg | 1.72 | 0.02 | 0.01 | 1.38 | 0.02 | 0.02 | 0.03 |
|  |  |  |  |  |  |  |  |
| 3.1 | 1.763 | 0.026 | 0.017 | 1.416 | 0.016 | 0.023 | 0.022 |
| 3.2 | 1.864 | 0.019 | 0.032 | 1.488 | 0.046 | 0.033 | 0.058 |
| 3.3 | 1.733 | 0.036 | 0.035 | 1.267 | 0.058 | 0.025 | 0.039 |
| Rep 3 avg | 1.79 | 0.03 | 0.03 | 1.39 | 0.04 | 0.03 | 0.04 |

| **Supplementary Table S2. Strains and plasmids used in this study** | | |
| --- | --- | --- |
| **Strain/plasmid** | **Characteristics** | **Source** |
| *P. aeruginosa strains* | | |
| mPAO1 | WT | lab collection |
| mPAO1 + pBADGR | WT complemented with pBADGR | This work |
| mPAO1 + pBADGR-PilB D388A | WT complemented with PilB D388A/*pilB A1163C* | This work |
| mPAO1 *pilA::Tn5* | ISphoA/hah insertion at position of 163 in *pilA* | (1) |
| mPAO1 *pilA::Tn5* + pBADGR | ISphoA/hah insertion of *pilA* complemented with pBADGr | This work |
| mPAO1 *pilA::Tn5* + pBADGR- *pilA* | ISphoA/hah insertion of *pilA* complemented with *pilA* | This work |
| mPAO1 *pilB* :: Tn5 | ISphoA/hah insertion at position of 411 in *pilB* | (1) |
| mPAO1 *pilB* :: Tn5 +pBADGr | ISphoA/hah insertion in *pilB* complemented with pBADGR | This work |
| mPAO1 *pilB* :: Tn5 +pBADGr-*pilB* | ISphoA/hah insertion in *pilB* complemented with *pilB* | This work |
| mPAO1 *pilB* :: Tn5 +pBADGr-PilB D388A | ISphoA/hah insertion in *pilB* complemented with PilB D388A | This work |
| mPAO1 PilB D388A +pBADGr | mPAO1 PilB D388A/*pilB A1163C* complemented with pBADGr | This work |
| mPAO1 PilB D388A +pBADGr-*pilB* | mPAO1 PilB D388A/*pilB A1163C* complemented with *pilB* | This work |
| mPAO1 *pilD^12^* | 12 base duplication of residues 548-559 of *pilD* | This work |
| mPAO1 *pilD^12^* + pBADGR | 12 base duplication of residues 548-559 of *pilD* complemented with pBADGR | This work |
| mPAO1 *pilD^12^* + pBADGR | 12 base duplication of residues 548-559 of *pilD* complemented with *pilA* | This work |
| mPAO1 *pilD^3^* | 3 base duplication of residues 548-550 of *pilD* | This work |
| mPAO1 *pilD^6^* | 6 base duplication of residues 548-553 of p*ilD* | This work |
| mPAO1 *pilD^9^* | 9 base duplication of residues 548-556 of *pilD* | This work |
| mPAO1 *pilD^12var^* | Insertion of TTTGGCGCCGTT following 548 of *pilD* | This work |
| mPAO1 *pilD::Frt* | ISphoA/hah insertion at position of 416 in *pilD* | (1) |
| mPAO1 *pilD::Tn5*/*pilA* '+pBADGR'-*pilA^chi^* | ISphoA/hah insertion of *pilD*, deletion of *pilA,* complemented with *pilA-hxcT chimera* | This work |
| mPAO1 *pilD^3^*/*pilA* '+pBADGR'-*pilA^chi^* | 3 base duplication of residues 548-550 of *pilD*, deletion of *pilA*, complemented with *pilA-hxcT chimera* | This work |
| mPAO1 *pilD^6^*/*pilA* '+pBADGR'- *pilA^chi^* | 6 base duplication of residues 548-553 of p*ilD*, pilA ISphoA/hah insertion at position 163 in *pilA*, complemented with pilA-hxcT chimera | This work |
| mPAO1 *pilD^9^/pilA* '+pBADGR'- *pilA^chi^* | 9 base duplication of residues 548-556 of *pilD, pilA ISphoA/hah insertion at position 163 in pilA, complemented with pilA-hxcT chimera* | This work |
| mPAO1 pilD^12^/pilA '+pBADGR'-*pilA^chi^* | 12 base duplication of residues 548-559 of *pilD, pilA ISphoA/hah insertion at position 163 in pilA, complemented with pilA-hxcT chimera* | This work |
| mPAO1 PilS N323A | Hyperactive *pilS A867G* mutant | (2) |
| mPAO1 PilS N323A | Hyperactive *pilS* mutant *A867G* complemented with pBADGR | This work |
| mPAO1 *pilD^12^/* PilS N323A | Hyperactive *pilS A867G* mutant and 12 base duplication of residues 548-559 of *pilD* | This work |
| mPAO1 *pilD^12^/* PilS N323A +pBADGr | Hyperactive *pilS A867G* mutant and 12 base duplication of residues 548-559 of *pilD* complemented with pBADGR | This work |
| mPAO1 *pilT::Tn5* | ISphoA/hah insertion at position of 885 in *pilT* | (1) |
| mPAO1 *pilD^12^/* *pilT::Tn5* | ISphoA/hah insertion in *pilT* 12 base duplication of residues 548-559 of *pilD* | This work |
| *E. coli strains* | | |
| DH5α | *F-φ80lacZΔM15 Δ(lacZYA-argF)U169 recA1 endA1 hsdR17(rk−, mk+) phoA supE44 thi-1 gyrA96 relA1 λ−* | Invitrogen |
| SM10 | *thi-1 thr leu tonA lacY supE recA::RP4-2-Tc::Mu (KmR)* | Invitrogen |
| Plasmids | | |
| pEX18Gm | Suicide vector for gene replacement | (3) |
| pBADGr | arabinose-inducible, broad host range complementation vector | (4) |
| pEX18Gm- *pilD^12^* | Construct to insert *pilD^12^* | This work |
| pEX18Gm- *pilD^3^* | Construct to insert *pilD^3^* | This work/IDT Gblock |
| pEX18Gm- *pilD6* | Construct to insert *pilD^6^* | This work/IDT Gblock |
| pEX18Gm- *pilD^9^* | Construct to insert *pilD^9^* | This work/IDT Gblock |
| pEX18Gm- *pilD^12var^* | Construct to insert *pilD12*var | This work/IDT Gblock |
| pEX18Gm-PilB D388A | Construct to insert PilB D388A | This work |
| pBADGR-*pilA^chi^* | Construct to complement *pilA-hxcT chimera* | This work |
| pBADGr-*pilA* | Construct to complement *pilA* | This work |
| pBADGr-*pilB* | Construct to complement *pilB* | This work |
| pBADGr-PilB D388A | Construct to complement PilB D388A | This work |

| **Supplementary Table S3. Primers used in this study** | | |
| --- | --- | --- |
| **Gene** | **Forward (5’ 🡪 3’)** | **Reverse (5’ 🡪 3’)** |
| Chromosomal knock in primers | | |
| *pilD^12^* | ACAAGAGCTCGAGTGGTGGCTTGCCG | CACAAAGCTTCAGCACGTCGTCGGC |
| *pilB* D388A | ACAAGAATTCATGAACGACAGCATCCAACTGAGC | ACAACCCGGGTTAATCCTTGGTCACGCGGTTGAC |
| Complementation primers | | |
| *pilA^chi^* | AATAGAATTCATGGATGTCGTGCAGTTCAGCTCCAGCCCGAAAGGACATCGGGGACAGCGGGGCTTTACCTTGATCGAA | TTGTTGGGCCCCGCATCGGCCTCTACGATG |
| *pilA* | AACAAGAGCTCCAAAGAGCTTGTTGCCGCG | TTGTTGGGCCCCGCATCGGCCTCTACGATG |
| *pilB* | ACAAGAATTCATGAACGACAGCATCCAACTGAGC | ACAACCCGGGTTAATCCTTGGTCACGCGGTTGAC |
| *pilB* D388A | ACAAGAATTCATGAACGACAGCATCCAACTGAGC | ACAACCCGGGTTAATCCTTGGTCACGCGGTTGAC |

**Supplementary References**

1. M. A. Jacobs, *et al.*, Comprehensive transposon mutant library of *Pseudomonas aeruginosa*. *Proc. Natl. Acad. Sci.* **100**, 14339–14344 (2003).

2. S. L. N. Kilmury, K. J. Graham, R. P. Lamers, L. T. MacNeil, L. L. Burrows, Hyperpiliation, not loss of pilus retraction, reduces *Pseudomonas aeruginosa*. *Microbiol. Spectr.* **13**, e02558-24.

3. T. T. Hoang, R. R. Karkhoff-Schweizer, A. J. Kutchma, H. P. Schweizer, A broad-host-range Flp-FRT recombination system for site-specific excision of chromosomally-located DNA sequences: application for isolation of unmarked Pseudomonas aeruginosa mutants. *Gene* **212**, 77–86 (1998).

4. M. L. Asikyan, J. V. Kus, L. L. Burrows, Novel Proteins That Modulate Type IV Pilus Retraction Dynamics in *Pseudomonas aeruginosa*. *J. Bacteriol.* **190**, 7022–7034 (2008).
